# The susceptibility of cochlear outer hair cells to cyclodextrin is not related to their electromotile activity

**DOI:** 10.1186/s40478-018-0599-9

**Published:** 2018-09-24

**Authors:** Yingjie Zhou, Satoe Takahashi, Kazuaki Homma, Chongwen Duan, Jason Zheng, Mary Ann Cheatham, Jing Zheng

**Affiliations:** 1 Department of Communication Sciences and Disorders, Northwestern University, Evanston, IL 60208 USA; 2 Department of Otolaryngology – Head and Neck Surgery, Feinberg School of Medicine, Northwestern University, Chicago, IL 60611 USA; 3 Knowles Hearing Center, Northwestern University, Evanston, IL 60208 USA

**Keywords:** Prestin, Niemann-pick type C1, HPβCD, Cholesterol, Salicylate, Electromotility, Outer hair cells

## Abstract

Niemann-Pick Type C1 (NPC1) disease is a fatal neurovisceral disorder caused by dysfunction of NPC1 protein, which plays a role in intracellular cholesterol trafficking. The cholesterol-chelating agent, 2-hydroxypropyl-β-cyclodextrin (HPβCD), is currently undergoing clinical trials for treatment of this disease. Though promising in alleviating neurological symptoms, HPβCD causes irreversible hearing loss in NPC1 patients and outer hair cell (OHC) death in animal models. We recently found that HPβCD-induced OHC death can be significantly alleviated in a mouse model lacking prestin, an OHC-specific motor protein required for the high sensitivity and sharp frequency selectivity of mammalian hearing. Since cholesterol status is known to influence prestin’s electromotility, we examined how prestin contributes to HPβCD-induced OHC death in the disease context using the NPC1 knockout (KO) mouse model (NPC1-KO). We found normal expression and localization of prestin in NPC1-KO OHCs. Whole-cell patch-clamp recordings revealed a significant depolarization of the voltage-operating point of prestin in NPC1-KO mice, suggesting reduced levels of cholesterol in the lateral membrane of OHCs that lack NPC1. OHC loss and elevated thresholds were found for high frequency regions in NPC1-KO mice, whose OHCs retained their sensitivity to HPβCD. To investigate whether prestin’s electromotile function contributes to HPβCD-induced OHC death, the prestin inhibitor salicylate was co-administered with HPβCD to WT and NPC1-KO mice. Neither oral nor intraperitoneal administration of salicylate mitigated HPβCD-induced OHC loss. To further determine the contribution of prestin’s electromotile function, a mouse model expressing a virtually nonelectromotile prestin protein (499-prestin) was subjected to HPβCD treatment. 499-prestin knockin mice showed no resistance to HPβCD-induced OHC loss. As 499-prestin maintains its ability to bind cholesterol, our data imply that HPβCD-induced OHC death is ascribed to the structural role of prestin in maintaining the OHC’s lateral membrane, rather than its motor function.

**Electronic supplementary material:**

The online version of this article (10.1186/s40478-018-0599-9) contains supplementary material, which is available to authorized users.

## Introduction

Cyclodextrins (CDs) comprise a family of amphipathic cyclic oligosaccharides with a hydrophobic core and a hydrophilic outer surface. Because CDs can complex with hydrophobic molecules to enhance their solubility, they have been widely used in the pharmaceutical industry to facilitate drug delivery [50]. More recently, CDs themselves have become attractive therapeutic candidates that can extract lipids and cholesterol from cell membranes to treat cardiovascular and neurodegenerative diseases [11]. One such example is 2-hydroxypropyl-β-cyclodextrin (HPβCD), which is currently undergoing a clinical trial for the treatment of a rare genetic disease called Niemann-Pick Type C1 (NPC1) [34, 35]. In NPC1 disease, lack or dysfunction of NPC1 protein that resides in endosomal and lysosomal membranes [27] disturbs intracellular cholesterol trafficking, resulting in progressive neural degeneration and early death in affected individuals. HPβCD administration drastically improves the neurological symptoms of both NPC1 animal models and human patients; however, hearing loss is reported to be a common outcome associated with HPβCD treatment [12, 13, 17, 22, 55].

Since hearing loss is an unavoidable adverse effect of HPβCD therapy in NPC1 patients, understanding the mechanisms underlying its ototoxicity is of high interest. In animal models, HPβCD-induced hearing loss is associated with loss of outer hair cells (OHCs) [13]. OHCs function as cochlear amplifiers by undergoing somatic length changes, called electromotility [5], which are required for sensitivity and frequency selectivity in mammalian hearing [15]. OHC electromotility is mediated by a distinctive motor protein, prestin [59], which belongs to a family of solute carrier protein 26 (SLC26) anion transporters. Prestin (SLC26A5) is unusual among the SLC26 family as it exhibits robust voltage-dependent conformational changes that confer electromotility. Recently, we discovered that HPβCD-induced OHC death is exacerbated by the presence of prestin, as OHCs lacking prestin were less vulnerable to HPβCD [45]. We further demonstrated that prestin can directly interact with cholesterol, suggesting that the extraction of cholesterol by HPβCD may disrupt the prestin-rich membrane, resulting in rapid OHC death [45]. Although prestin contributes to the OHC’s sensitivity to cholesterol depletion, it is not clear how prestin influences HPβCD-evoked OHC death or whether prestin’s motile function contributes to its vulnerability to HPβCD treatment.

The OHC’s lateral membrane is packed with ~ 11 nm particles composed of prestin tetramers [25, 54, 58]. In fact, removing prestin results in a 40% decrease in OHC length [8], indicating that cholesterol concentration and the lipid environment are probably wild-type (WT)-like, as cell surface area decreases proportionally in OHCs lacking prestin. Although cholesterol molecules bound to WT prestin could be the reason that OHCs are more sensitive to HPβCD treatment, we cannot rule out the possibility that prestin’s motile function may also contribute to susceptibility, as cholesterol is known to influence both motility and oligomerization [21, 37]. Prestin’s motile function is based on its ability to change its conformation when membrane voltage is changed. As β-cyclodextrin is capable of changing protein conformation [18], whether prestin’s motile function contributes to its vulnerability to HPβCD treatment requires investigation.

In this study, we sought to understand the molecular mechanisms underlying HPβCD-induced OHC death in the disease context. NPC1 patients present with variable degrees of hearing impairment even before receiving their first HPβCD treatment [24]. The NPC1-knockout (KO) mouse model (also known as *NPC*^*nih*^) that lacks NPC1 expression exhibits hearing impairment well before the onset of overt neurological symptoms [23]. These observations suggest that NPC1 plays important roles directly or indirectly in hearing, as decreased OHC performance was detected by measuring distortion product otoacoustic emissions (DPOAE). Since cholesterol has an enormous influence on prestin’s function and structure [21, 37], we first tested whether the expression, localization, and/or function of prestin is influenced in the NPC1 disease context using the NPC1-KO mouse model. Our data show that lack of NPC1 protein does not affect the normal distribution pattern of prestin protein. Using an electrophysiological method, we assessed function of prestin by measuring nonlinear capacitance (NLC), a proxy for electromotility. OHCs isolated from NPC1-KOs exhibited robust NLC indicating that prestin-based somatic electromotility is present, although its sensitivity and voltage dependence are altered relative to WT prestin. Consistent with normal prestin expression, OHCs from NPC1-KOs are as sensitive to HPβCD as WT. In order to determine whether the motile function of prestin contributes to the HPβCD-induced ototoxicity, we utilized the prestin inhibitor salicylate, a commonly used painkiller and anti-inflammatory drug known as aspirin. Salicylate competes with prestin’s substrates such as chloride and bicarbonate, thereby reversibly inhibiting function [33]. Co-administration of salicylate and HPβCD did not mitigate HPβCD-induced OHC death, indicating that inhibition of prestin’s electromotility did not affect the sensitivity of OHCs to HPβCD. We further tested the contribution of prestin’s motile function using a prestin knockin (KI) mouse model that expresses virtually nonfunctional 499-prestin protein (499-prestin-KI) [16]. 499-prestin KI mice were as sensitive to HPβCD-induced OHC loss as WT, suggesting that prestin’s motor action is not the key factor underlying the OHC’s sensitivity to HPβCD. Since 499-prestin targets the lateral membrane and interacts with cholesterol as in WT prestin, OHC loss appears to be determined by the presence of cholesterol-interacting prestin proteins that confer normal OHC stiffness and length, rather than to its electromotile function.

## Material and methods

### Animals

All experimental procedures were conducted in accordance with the Guide for the Care and Use of Laboratory Animals, and were approved by Northwestern University’s Animal Care and Use Committee and the National Institutes of Health. NPC1-KO mice (BALB/cNctr-*Npc1*^*m1N*^, also known as *NPC*^*nih*^) were obtained from The Jackson Laboratory (Stock No: 003092). Wild-type (WT) and NPC1-KO mice were obtained by heterozygous breeding. Genotyping was outsourced to Transnetyx (Cordova, TN). Mice younger than 2.5 months (age) were used to avoid complications from neurological dysfunction due to loss of NPC1. 499-prestin-KI mice that carry the V499G/Y501H mutation in the *prestin* gene were maintained on the original 129S6/C57Bl6J background [16]. 499-KI mice younger than 1 month of age were used to minimize OHC loss [8]. In all experiments, both males and females were tested.

### HPβCD and HPβCD/salicylate treatments

WT and 499-prestin-KI mice were injected with saline or HPβCD (Sigma, H107) dissolved in saline (0.9% NaCl) subcutaneously as described previously [45]. For low-dose (4000 mg/kg) treatment, mice were repeatedly injected four times once per week for four weeks. For high-dose (8000 mg/kg) treatment, mice were injected once. For oral administration of salicylate, adult mice (P32–43) were supplied with drinking water containing 3 mg/ml sodium salicylate for 7 days prior to high-dose HPβCD subcutaneous injection. Salicylate water bottles were changed twice per week. Animals were returned to regular water 24 h after HPβCD injection. For intraperitoneal administration of salicylate, mice (P32–45) were first injected with sodium salicylate (245 mg/kg) a day before HPβCD treatment. Approximately, 16 h later, a second salicylate injection was administered as described before [57]. Within 1 h, mice were screened using DPOAEs and auditory brainstem responses (ABR), and then injected with 8000 mg/kg HPβCD subcutaneously, i.e., ~ 1 h after the second salicylate injection. Since salicylate is known to be eliminated from the blood after 8 h in mice [44], these animals were also supplied with water containing 3 mg/ml sodium salicylate, but returned to regular water 24 h after HPβCD injection. Control mice were supplied with regular water and injected with saline. Auditory function was measured before and after HPβCD injection.

### Cochlear in vivo physiology

WT and NPC1-KO mice were anaesthetized with intraperitoneal injections of ketamine (100 mg kg^− 1^) and xylazine (10 mg kg^− 1^). Additional doses were given throughout the experiment to maintain a surgical level of anesthesia. During data collection, body temperature was maintained using a heating blanket. DPOAE were measured by presenting the two stimulating primaries (f2/f1 = 1.2) at 70 dB SPL or when the level of f1 was 50 dB SPL and the level of f2 as 35 dB SPL. Growth or input-output functions were also acquired for f2 = 12 kHz and for f2 = 27 kHz. For these measurements, the level of f1 was always 10 dB higher than that for f2. In order to obtain a measure of sensitivity, DPOAE thresholds were determined as the level of f1 that generated 2f1-f2 of 0 dB. ABRs to tone-burst stimuli were then collected to document the output of the cochlea, as well as the brainstem. For these recordings, subcutaneous electrodes were inserted at the vertex and the mastoid with each response measured relative to the indifferent electrode inserted into the opposite shoulder/neck region. Calibration was performed quasi-free field as reported previously [36]. Thresholds were determined by noting the signal level where the ABR waveform disappeared into the noise. Because the number of averages increased as signal level decreased (5 dB step size), the noise level was ~ 0.2 microvolts. Further details on how these recordings are made appear in a previous publication [9]. All DPOAE and ABR measurements were completed on the same day.

### Nonlinear capacitance (NLC) measurements

OHCs were isolated from P29–78 mice for NLC recordings. Whole-cell recordings were performed at room temperature using the Axopatch 200A amplifier (Molecular Devices, CA). Recording pipettes were pulled from borosilicate glass to achieve initial bath resistances averaging 3~ 4 MΩ. Intracellular pressure was kept at 0 mmHg during recording. Recording pipettes were filled with an intracellular solution containing (mM): 140 CsCl, 2 MgCl2, 10 EGTA, and 10 HEPES (pH 7.3). OHCs were isolated from WT and NPC-KO mice and bathed in an extracellular solution containing (mM): 120 NaCl, 20 TEA-Cl, 2 CoCl2, 2 MgCl2, 10 HEPES (pH 7.3). Osmolarity was adjusted to 310 mmol/kg with glucose. NLC was measured using a sinusoidal voltage stimulus (2.5-Hz, 120 or 150 mV amplitude) upon which two higher frequency stimuli were superimposed (391 and 781 Hz, 10 mV amplitude). Data were collected by jClamp (SciSoft Company, New Haven, CT), and NLC determined, as described previously [19, 41]. Voltage-dependent cell membrane electrical capacitance data were analyzed using the following two-state Boltzmann equation:

$$ {C}_m=\frac{\alpha {Q}_{\mathrm{max}}\exp \left[\alpha \left({V}_m-{V}_{pkcm}\right)\right]}{{\left\{1+\exp \left[\alpha \left({V}_m-{V}_{pkcm}\right)\right]\right\}}^2}+{C}_{lin} $$

where α is the slope factor of the voltage-dependence of charge transfer, Q_max_ is the maximum charge transfer, V_m_ is the membrane potential, V_pkcm_ is the voltage at which the maximum charge movement is attained, and C_lin_ is the linear capacitance [20].

### Immunostaining and anatomical measurements

Mice were cardiac perfused with 4% paraformaldehyde and cochleae extracted. After post-fixation and decalcification, cochleae were dissected following the Eaton-Peabody Laboratory cochlear dissection protocol [28]. In order to detect prestin, N-terminal prestin rabbit antisera [58] was used at 1:1000 with goat anti-rabbit Alexa Fluor 488 (Thermo) as the secondary antibody at 1:500. Alexa 546-conjugated phalloidin and Hoechst 33342 (Thermo) were also used to stain actin and nuclei, respectively, as described before [45]. Stained cochlear sections were mounted onto slides using Dako fluorescent mounting medium (Agilent). Images were captured on a Nikon A1R confocal microscope with Plan Fluor 10X and Plan Apo 20X objectives (Nikon) controlled by NIS Element software. Basilar membrane length was measured using ImageJ, and the numbers of remaining OHCs determined. A mouse cochlear place-frequency map [32] was used to determine the corresponding frequencies.

### Plasmids, cell line and cell culture

To generate pIZ-499-prestin-ceGFP, pIZ-gPres-ceGFP that contained full-length gerbil prestin with the C-terminal V5 and GFP tag in the pIZ-V5/His vector (Thermo Fisher) [45] was mutagenized using QuickChange Site-Directed Mutagenesis Kit (Agilent) following the manufacturer’s instructions. To introduce 499 mutations (V499G/Y501H) [58], the following primers were used: gPres V499A/Y501H A (5′- CATTGCTCTGCTGACTGGGATCCACAGAACCCAGAGTCC -3′) and gPres V499A/Y501H B (5′- GGACTCTGGGTTCTGTGGATCCCAGTCAGCAGAGCAATG -3′). Sf9 cells (Thermo Fisher) were maintained in Sf-900 III SFM supplemented with 5% fetal bovine serum (Gibco) and 1X antibiotic antimycotic solution (Sigma). To generate stable Sf9 cells expressing 499-prestin protein, Sf9 cells were transfected with pIZ-499-prestin-V5_ceGFP using Effectene (Qiagen), and selected with 1 μg/μl zeocin (Thermo Fisher). A single clone was chosen to establish the stable cell line. Generation of the stable sf9-prestin-ceGFP cell line, which expresses WT prestin, was previously reported [45].

### Cholesterol binding assay

Pre-washed CarboxyLink coupling gel was processed with or without cholesteryl hemisuccinate to prepare cholesterol-beads and unconjugated control-beads for non-specific binding as described before [45]. Cell lysates containing membrane fractions isolated from stable Sf9 cells expressing WT-prestin and 499-prestin were mixed with cholesterol-beads or unconjugated control beads and incubated for 1 h at room temperature. The reaction mix was then centrifuged and washed 5 times with 50 mM Tris-Cl (pH 7.5) buffer containing 50 mM NaCl and 20 mM DDM (n-dodecyl β-D-maltoside), and eluted with 2X Laemmli Sample Buffer (BioRad). Eluates were analyzed by Western blotting as described [45].

## Results

### Preservation of prestin expression and motor function in OHCs of NPC1-KO mice

**Fig. 1:**
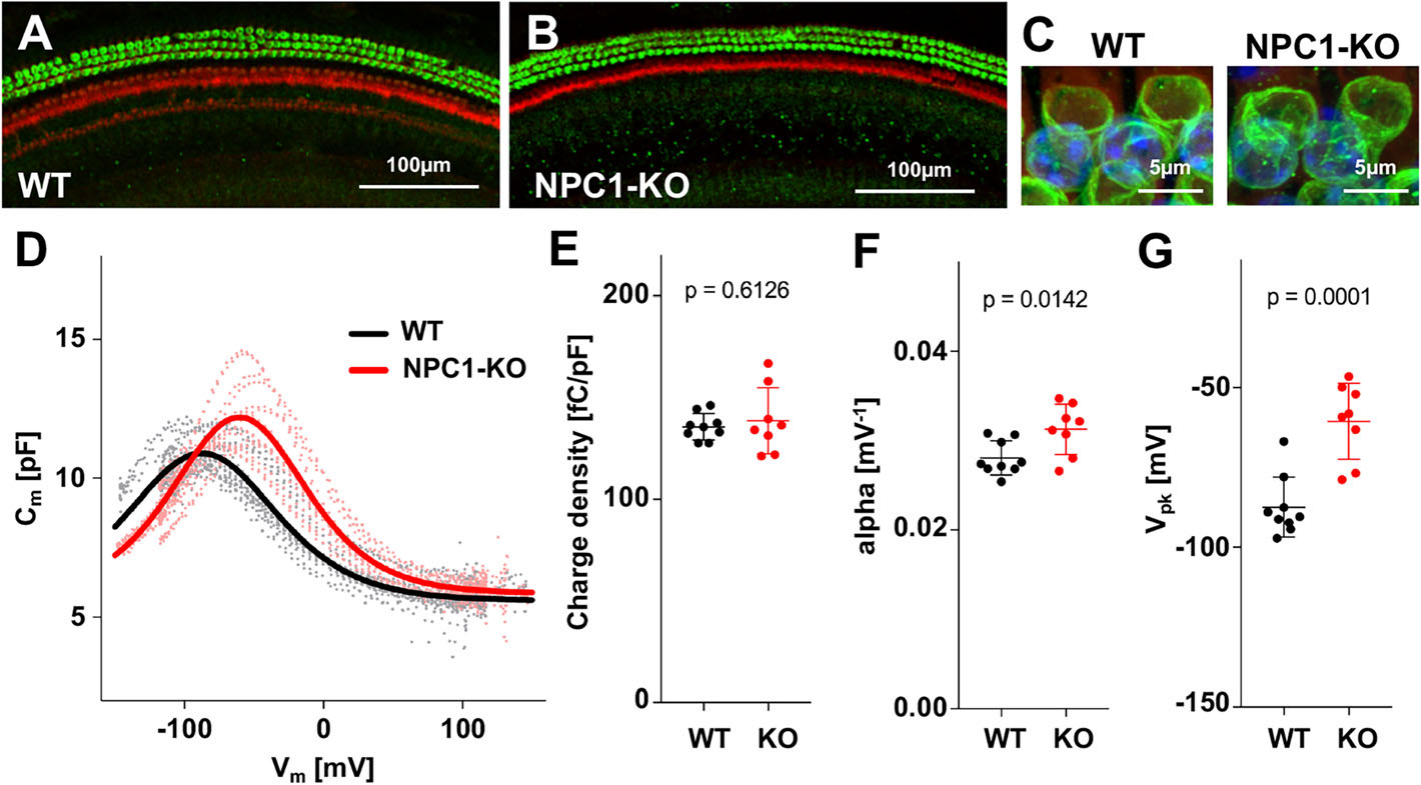
Prestin expression and function in NPC1-KO mice. **a-b** Immunofluorescent images of OHCs from WT and NPC1-KO mice showing normal prestin expression. Representative images from the middle of the cochlea in a P49 female WT (**a**) and P49 male NPC1-KO (**b**), stained with anti-N-mprestin (green) and phalloidin-Alexa 546 (red) showing normal prestin expression. Scale bars, 100 μm. **c.** 3D reconstruction of z-stack images of OHCs from P49 male WT and female NPC1-KO cochleae (scale bar, 5 μm), showing the characteristic cylindrical staining of prestin at the lateral membrane. Red, phalloidin (actin); Blue, nuclei. **d-g.** NLC measurements of OHCs isolated from two female P52 WT and one female P78 and two male P29 NPC1-KO mice. **d** Individual recordings of NLC are shown in gray (WT) and pink (NPC1-KO), and the solid black (WT) and red (NPC1-KO) lines indicate fitted curves. **e** Charge density (CD). Mean ± s.d. are: WT, 135.5 ± 6.5 fC/pF (*n* = 9); NPC1-KO, 138.6 ± 16.2 fC/pF (*n* = 8). ***F*** *alpha*. Mean ± s.d. are: WT, 0.028 ± 0.002 mV^− 1^ (*n* = 9); NPC1-KO, 0.031 ± 0.003 mV^− 1^ (*n* = 8). **g** V_pkcm_. Mean ± s.d. are: WT, − 87.41 ± 9.3 mV (*n* = 9); NPC1-KO, − 60.57 ± 12.0 mV (*n* = 8). *P* values are indicated in each figure (unpaired t-test)

Niemann-Pick Disease Type C1 is a fatal genetic neurovisceral disorder characterized by a failure to traffic intracellular cholesterol [51]. It is also known that the metabolic status of cholesterol in NPC1-KO mice influences gene and protein expression patterns [10]. Since cholesterol can affect prestin oligomerization, localization to the lateral membrane, and lipid raft association [37, 38], we examined whether prestin is properly expressed and localized in OHCs of NPC1-KO mice. As shown in Fig. 1a-b, OHCs appear normal in cochlear samples from NPC1-KOs (*n* = 3) with prestin expression patterns similar to WT (*n* = 2). Like WT, prestin localizes exclusively at the lateral membrane, suggesting that lack of NPC1 protein does not influence prestin-expression in OHCs (Fig. 1c).

To evaluate prestin’s electromotility, we measured and compared nonlinear capacitance (NLC) of OHCs isolated from WT and NPC1-KO mice. Figures 1d-g summarize the NLC measurements, which provide a signature of prestin’s motor activity [2, 40]. There was no statistically significant difference in the charge density (Fig. 1e) between WT and NPC1-KO. This parameter, charge density (CD, defined as Q_max_/ C_lin_), normalizes the amount of prestin activity to cell size, such that Q_max_ correlates with the amount of functional prestin expressed in the cell membrane, and C_lin_ is an indication of OHC size. These data are consistent with the staining results, indicating that similar amounts of prestin protein were expressed in adult WT and NPC1-KO OHCs. We did, however, observe significant changes in alpha (Fig. 1f), which indicates voltage sensitivity, as well as a depolarizing shift in V_pkcm_ (Fig. 1g). These observations are consistent with previous reports showing that cholesterol affects the sensitivity of prestin, and decreasing cholesterol in the membrane shifts V_pkcm_ in the depolarizing direction [21, 37, 45]. Hence, the fact that OHCs from NPC1-KO mice have a more depolarized V_pkcm_ suggests that the cholesterol content of the membrane in NPC1-KO mice could be lower than that of OHCs from WT mice [52]. Taken together, lack of NPC1 does not affect prestin expression and membrane targeting. However, prestin’s sensitivity and V_pkcm_ are altered, which could relate to a reduction in the cholesterol content of the OHC’s plasma membrane in NPC1-KO mice.

### Reduced sensitivity and OHC loss in NPC1-KO mice

**Fig. 2:**
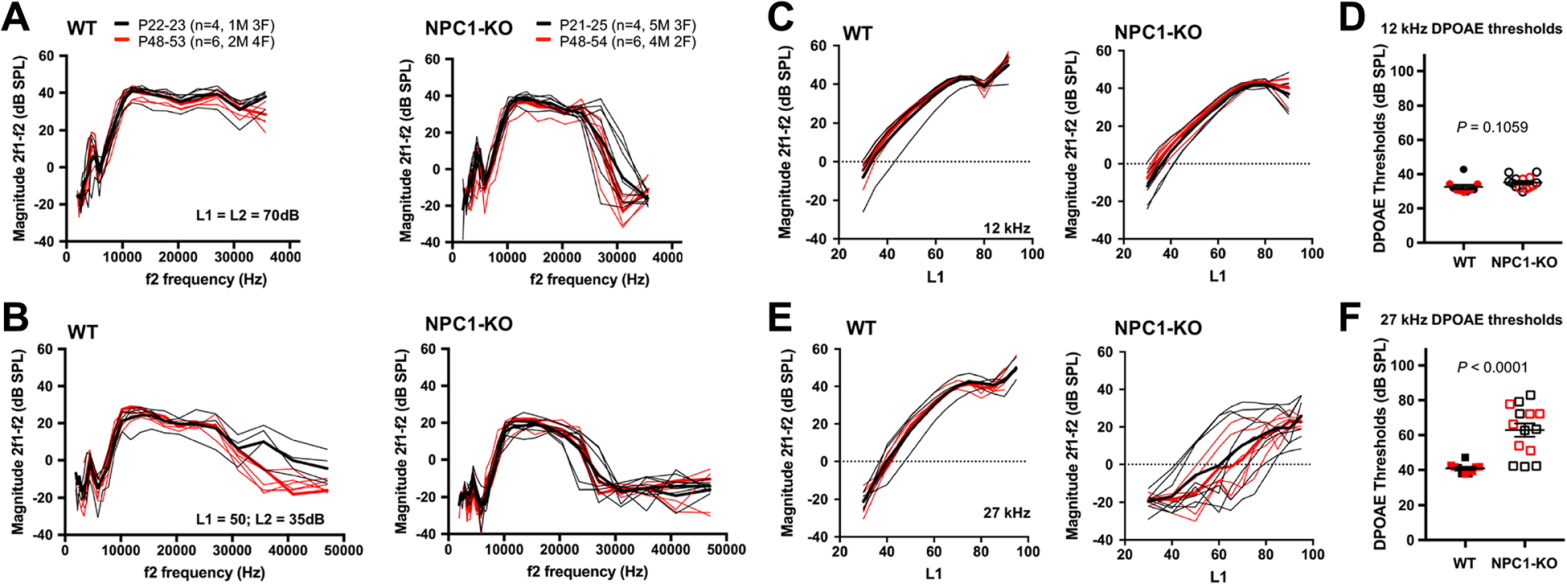
In vivo physiology of WT and NPC1-KO mice. **a-b** DP-grams (f2/f1 = 1.2) for mean 2f1–f2 at high (L1 = L2 = 70 dB, **a**) and moderate (L1 = 50 dB; L2 = 35 dB, **b**) stimulus levels for WT and NPC1-KO mice at P21–25 (black lines) and P48–54 (red lines). Numbers (n) and sexes of the mice (M, male; F, female) are also indicated. Thin black and red lines are individual traces. In 2B, responses at f2 = 40,910 and 35,625 Hz for older (48–54) mice were significantly reduced (*p* = 0.03 and 0.0063, respectively) as compared to younger (P21–25) animals. **c-f** DPOAE input-output functions collected when the level for f1 is 10 dB higher than that for f2 at f2 = 12 kHz (**c-d**), and f2 = 27 kHz (**e-f**). DPOAE thresholds for 12 kHz (**d**) and 27 kHz (**f**) are also plotted based on the input-output functions in (**c**) and (**e**), respectively. Threshold is defined as the level of f1 that generates a DPOAE at 2f1-f2 of 0 dB. The black color indicates data points from P21–25 mice while the red color designates P48–54 mice. At f2 = 27 kHz, DPOAE thresholds of NPC1-KO mice are significantly elevated compared to WT

Prestin provides the molecular basis for OHC somatic electromotility [5, 59] and is essential for normal hearing [16, 29]. To investigate whether the function of OHCs in NPC1-KO mice is affected by lack of NPC1 in vivo, we compared DPOAEs in WT and NPC1-KO mice, since this metric is associated with OHC integrity [3]. As shown in Fig. 2, DPOAE magnitudes in NPC1-KO mice are similar to WT littermates at low frequencies at P21-P54 (Fig. 2a-d). In contrast and similar to previous results [23], reduced sensitivity is observed at high frequencies (27 kHz) in both male and female mice. In fact, the WT results in Fig. 2b show a statistically significant reduction in DPOAE magnitudes at high f2 frequencies in the older cohort (red, P48-P53), consistent with the known age-related hearing loss in the albino BALB/c strain [56]. These changes are exacerbated in mice lacking NPC1 where magnitudes are reduced for f2 frequencies above ~ 25 kHz. To assay changes in sensitivity, growth functions for the DPOAE at 2f1-f2 are provided in panels C and E. Again, the results for f2 = 12 kHz are WT-like in mice lacking NPC1, where DPOAE thresholds (Fig. 2d) are similar to controls. However, the thresholds for f2 = 27 kHz are raised and highly variable in the NPC1-KO animals (Fig. 2e-f).

**Fig. 3:**
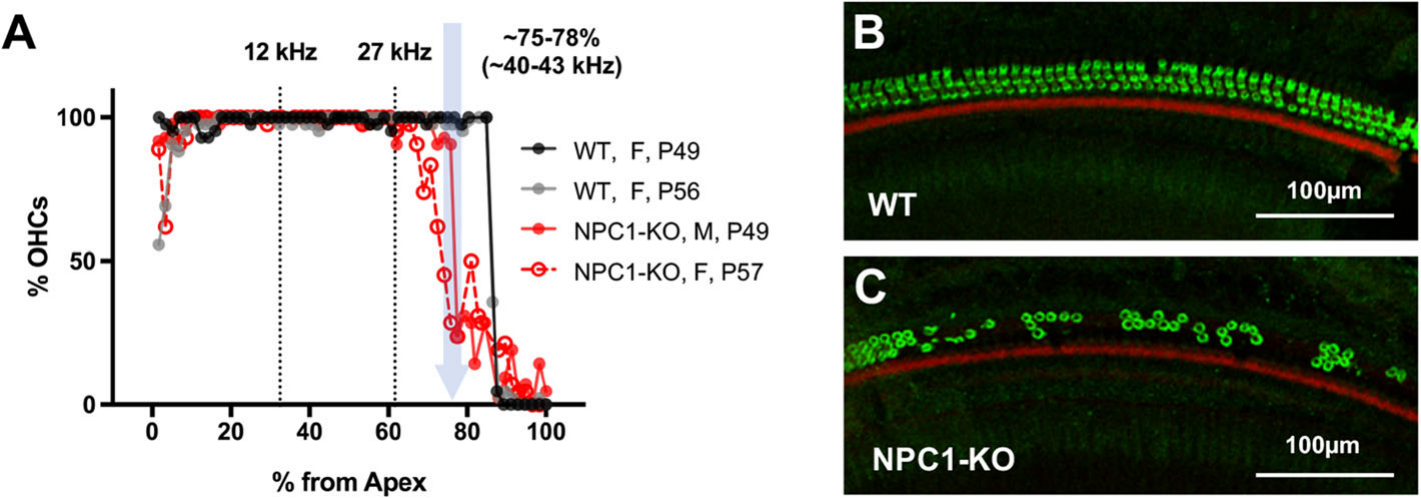
OHC loss in the basal region of the cochlea in NPC1-KO mice. **a**. Cytocochleograms of WT and NPC1-KO littermates from a heterozygous breeder pair, where the % OHC survival is plotted along the length of cochlea. Vertical black dotted lines indicate the regions corresponding to 12 and 27 kHz. **b-c** Immunofluorescent images showing the basal region (~ 75% of the distance from the apex, as indicated by the pale blue arrow in (**a**) for a P56 female WT (**b**) and a P49 male NPC1-KO (**c**) In this region, scattered OHC loss is observed in NPC1-KO but not in WT. Whole-mount OC sections were stained with anti-N-mprestin antibody (green) and phalloidin-Alexa 546 (red). Scale bars, 100 μm

To evaluate OHC preservation in NPC1-KO mice, cytocochleograms were constructed for two cochleae from NPC1-KO mice that showed reduced sensitivity at high frequencies. OHC loss was observed in NPC1-KO mice in the basal region, starting around 68~ 75% of the distance from the apex, which is slightly above the 27 kHz place (61.6%) [32]. OHC loss in WT (*n* = 2) was not observed until ~ 85% of the distance from the apex (Fig. 3a). As shown in Fig. 3b-c, more than 50% of the OHCs were lost in the region located ~ 75% of the distance from the apex in NPC1-KO, while OHCs were largely intact in the WT control at the same position. Taken together, apical OHCs in NPC1-KO mice are functionally normal in vivo, while the loss of sensitivity at high-frequencies is at least in part due to the loss of OHCs at the base of cochlea. The fact that WT controls on the BALB/c background also had reduced DPOAEs at high f2 frequencies, indicates that OHCs just apical to the lesion boundary may not be functional although still present.

### *OHCs from NPC1-KO mice are still sensitive to* HPβCD

**Fig. 4:**
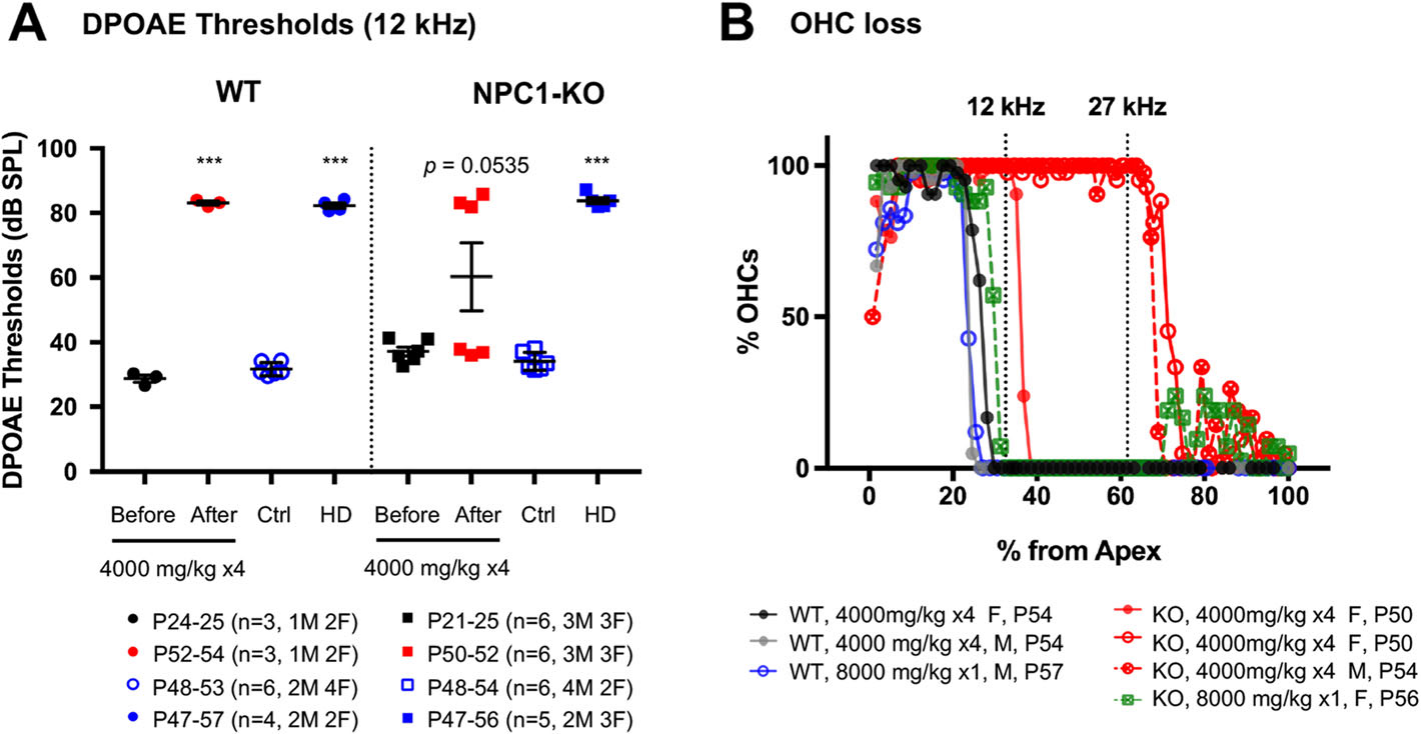
OHCs of NPC1-KO mice remain susceptible to HPβCD. **a** DPOAE thresholds at f2 = 12 kHz of WT and NPC1-KO mice before and after four weekly injections of 4000 mg/kg HPβCD are plotted. Mice receiving single injections of high-dose (HD) HPβCD at 8000 mg/kg and their age-matched controls (Ctrl) are also shown. Each dot represents one mouse. Numbers and sexes are also indicated. Statistical significance is indicated by three asterisks (*p* < 0.001, two-tailed t-test). Variations in sensitivity to low-dose HPβCD in NPC1-KO (“After”) is not sex-dependent. **b** Cytocochleograms of WT and NPC1-KO mice that received four injections of 4000 mg/kg HPβCD or single injection of 8000 mg/kg HPβCD from (**a**) NPC1-KO mice receiving the low dose HPβCD injections showed large variations in OHC loss that correspond to the DPOAE thresholds. NPC1-KO mice treated with high dose HPβCD exhibit greater OHC loss than the low-dose groups

HPβCD is more effective in reversing NPC symptoms in the NPC1 KO mice when delivered directly to the brain because HPβCD does not effectively cross the blood-brain barrier (BBB) [6]. For example, a 23 mg/kg HPβCD intracerebroventricular injection has a better outcome than a 4000 mg/kg HPβCD subcutaneous injection [1]. Similar to BBB, the blood labyrinthine barrier (BLB) in the cochlea also limits chemical transportation between blood and cochlear fluids. Using a clinical-grade LC/MSMS method of quantifying HPβCD, Crumling and colleagues found that the concentration of HPβCD detected in the cochlear fluids 3 h after 8000 mg/kg injection was ~ 128 μM, far below typical cytotoxic levels of HPβCD [14], and in the range suggested for treating NPC patients: < 1 mM or 100–400 μM [1, 31]. Previously, injections of 4000 mg/kg/week of HPβCD have been shown to increase the life span of NPC1-KO mice but cause loss of sensitivity [48, 53, 55]. Since HPβCD selectively kills OHCs in WT mice and produces threshold shifts, we examined whether this is also the case for NPC1-KO mice. First, we injected 4000 mg/kg/week to WT controls and their NPC1-KO littermates, starting after weaning (~P21) for 4 weeks and measured DPOAEs and ABRs before the first (“Before”) and after the last injection (“After”). Because of the variability of the high-frequency responses for NPC1-KO mice on the BALB/c background, we evaluated DPOAE thresholds at f2 = 12 kHz before or after HPβCD injections (4000 mg/kg × 4). As shown in Fig. 4a, DPOAE thresholds at 12 kHz were significantly increased by HPβCD treatment (4000 mg/kg × 4) in ***all*** WT mice tested. In addition, the in vivo measurements also showed loss of DPOAEs at all f2 frequencies (Additional file 1: Figure S1). As expected, this change in phenotype is due to the loss of OHC function, as anatomical examination of the HPβCD-treated WT mice showed massive OHC loss with only a few OHCs remaining near apex (black and gray lines, Fig. 4b). In contrast, responses in NPC1-KO mice were binary: three out of six animals (two males and one female) exhibited threshold shifts as in WT mice, whereas the other three mice (1 male and two females) did not show any change in DPOAE thresholds at 12 kHz for the 4000 mg/kg × 4 injections. Thus, variations in sensitivity to low-dose HPβCD in NPC1-KOs (“After”) are not sex-dependent. Consistent with this result, HPβCD-treated NPC1-KO mice with normal thresholds retained most of their OHCs (Fig. 4b, red open circles with solid lines and crossed circles with red broken lines), similar to cytocochleograms of NPC1-KO mice without HPβCD treatment (Fig. 3a, red lines). In contrast, HPβCD-treated NPC1-KO mice with increased DPOAE thresholds exhibited massive OHC loss (Fig. 4b, closed red circles and solid lines).

Since some NPC1-KO mice were resistant to HPβCD-induced OHC loss and reductions in DPOAEs, we increased the HPβCD dosage to a single administration of 8000 mg/kg, which is known to cause OHC death within hours [45]. As shown in Fig. 4a, a single injection of 8000 mg/kg HPβCD (HD for high dose) caused a statistically significant threshold shift in both WT and NPC-KO mice compared to their corresponding untreated groups (Ctrl) (Fig. 4a, *p* < 0.001). Although this particular NPC1-KO mouse retained slightly more OHCs with 8000 mg/kg HPβCD (Fig. 4b, green) than WT littermates (Fig. 4b, blue), this animal still suffered a vast reduction in the overall numbers of surviving OHCs. Taken together, NPC1-KO mice remain susceptible to HPβCD-induced OHC loss but exhibit large variations in their sensitivity to the low dosage of HPβCD when compared to WT.

### Co-administration of salicylate with HPβCD did not mitigate HPβCD-induced loss of sensitivity

**Fig. 5:**
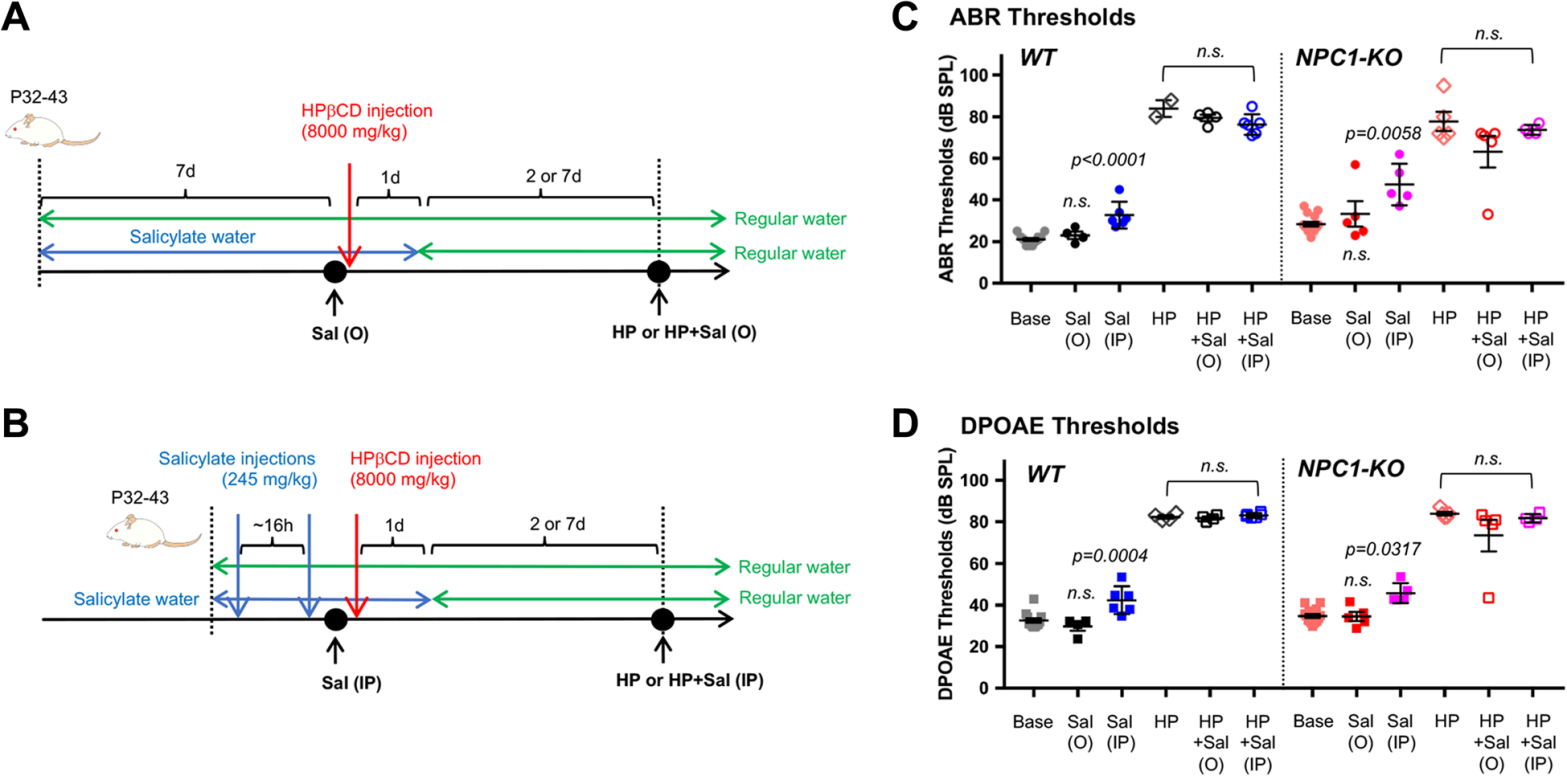
Salicylate treatment of WT and NPC1-KO mice did not mitigate HPβCD-induced threshold shifts. **a-b** Schematic representation of the protocol for oral administration (**a**) and intraperitoneal injection (**b**) of salicylate. **c-d** ABR (**c**) and DPOAE (**d**) thresholds of WT and NPC1-KO mice at 12 kHz before and after high-dose (8000 mg/kg) HPβCD injection. Base, baseline controls; HP, regular water, 8000 mg/kg HPβCD injection; Sal (O), 3 mg/ml salicylate water for 7 days before HPβCD injection; HP + Sal (O), 3 mg/ml salicylate water for 7 days before and 1 day after HPβCD injection; Sal (IP) 245 mg/kg salicylate injections; HP + Sal (IP), 245 mg/kg salicylate injections before HPβCD injection. For WT, Base: *n* = 12 (P22–53); HP: *n* = 2 for ABR (1 male 1 female), *n* = 4 for DPOAE (2 males, 2 females, P47–57); Sal (O): *n* = 4 (2 males, 2 females; P50); HP + Sal (O): *n* = 4 (2 males, 2 females; P52). For NPC1-KO, Base: *n* = 16 (P21–54); HP: *n* = 4 (2 males, 2 females; P47–57); Sal (O): *n* = 5 (2 males 3 females; *P* = 39–41); Sal (IP): *n* = 5 (3 males 2 females; *P* = 31–47); HP + Sal (O): *n* = 5 (2 males 3 females; P41–48); HP + Sal (IP): *n* = 4 (2 males 2 females; P33–49). One-way ANOVA with Tukey’s post analysis showed no significant (n.s.) difference between Base vs. Sal (O) or HP vs. HP + Sal (O) or HP + Sal (IP) groups in both WT and NPC1-KO; Sal (IP) groups in WT and NPC1-KO showed statistically significant threshold shifts as compared to Base (P values as indicated)

Since prestin is one of the key determinants of HPβCD-induced OHC death, we asked whether the motile function of prestin plays a role. Salicylate, commonly known as aspirin, is a small molecule inhibitor of prestin’s electromotility. It is well documented that salicylate reversibly inhibits OHC function and induces temporary hearing loss by directly interacting with prestin [33, 42]. Thus, salicylate may mitigate HPβCD-induced OHC death by inhibiting prestin’s electromotile function, should it be involved. To test this hypothesis, we co-administered salicylate with HPβCD to both WT and NPC1-KO mice and evaluated their auditory function and the degrees of OHC loss. We employed two modes of salicylate administration, oral (3 mg/ml salicylate in drinking water, “Sal (O)” in Fig. 5a) and intraperitoneal injection (245 mg/kg, “Sal (IP)” in Fig. 5b), either alone or in combination with high-dose HPβCD (8000 mg/kg, single injection, “HP” in Fig. 5a-b). DPOAEs and ABRs were measured at the time points outlined in Fig. 5a-b (Black dots). Since salicylate is known to be metabolized within 8 h in mice [44], the Sal (IP) group was also supplied with salicylate-containing water (3 mg/ml) for the duration of time indicated (Fig. 5b). For both WT and NPC1-KO, Sal (IP) groups exhibited slight but significant increase in ABR (Fig. 5c) and DPOAE (Fig. 5d, Additional file 1: Figure S2) thresholds as compared to the base line controls before the HPβCD injection (Base vs. Sal (IP)). This is consistent with the fact that high-dose salicylate treatment can cause reversible hearing loss [42]. However, this increase in ABR or DPOAE thresholds was not observed for the mice in Sal (O) groups (Fig. 5c-d, Base vs. Sal (O)). These results indicate that our protocol for oral administration of salicylate was not sufficient to influence prestin function in the cochlea. Consequently, threshold shifts induced by HPβCD were not mitigated in mice receiving salicylate in the drinking water for both WT and NPC1-KO groups (Fig. 5c-d, HP vs HP + Sal (O)). This was also the case for mice receiving salicylate by IP injections even though salicylate injections before HPβCD injection caused high-frequency ABR threshold shifts and presumably inhibited prestin function (Fig. 5c-d, HP vs. HP + Sal (IP)). According to Yu and his colleagues [57], DPOAEs recovered in ~ 8 h, i.e., OHC function was initially inhibited by salicylate. In addition, HPβCD reaches the cochlea in ~ 2 h and damages ~ 85% of OHCs within 8 h after a single 8000 mg/kg subcutaneous (SC) injection [14, 45]. Taken together, these results suggest that HPβCD-induced ototoxicity is not dependent on prestin’s motor function, as inhibition of prestin’s electromotility by salicylate did not affect the outcome of HPβCD-induced ototoxicity in either WT or NPC1-KO.

### HP βCD-induced ototoxicity does not depend on OHC electromotility

**Fig. 6:**
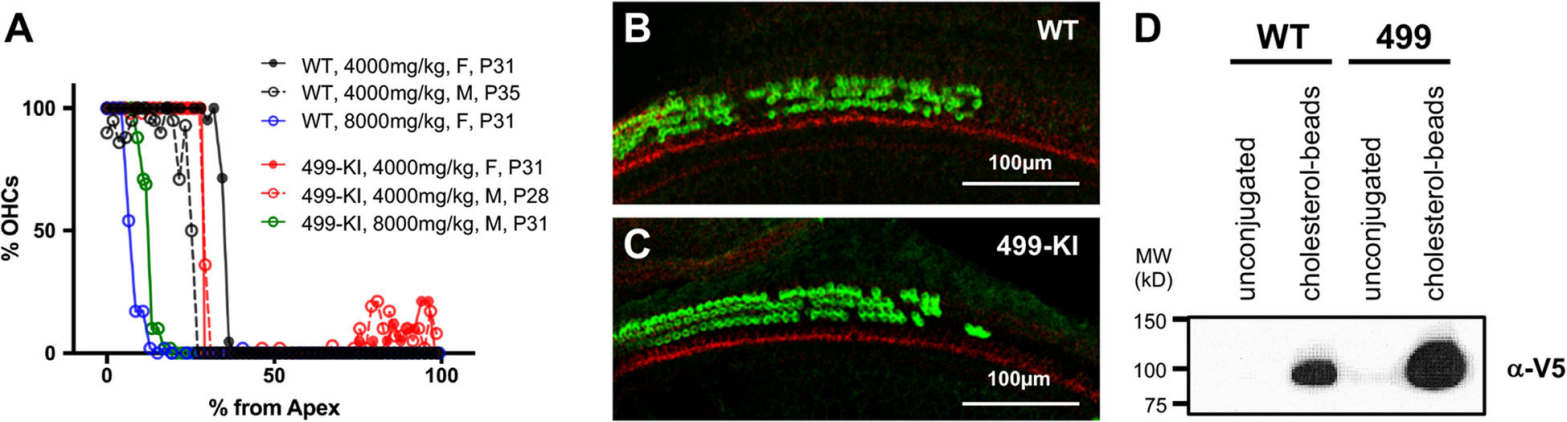
Non-motile OHCs are still sensitive to HPβCD. **a** Cytocochleograms of WT and 499-prestin-KI mice after HPβCD treatment (4000 mg/kg or 8000 mg/kg). Age and sex of mice are indicated. **b-c**. Immunofluorescent images of the OC from a P35 male WT (**b**) and a P28 male 499-prestin-KI (**c**) one week after a single 4000 mg/kg HPβCD injection. Images were obtained ~ 27% of the distance from apex to show the beginning of the OHC lesion boundary. Cochlear whole mount sections were stained with anti-N-mprestin antibody (green) and phalloidin-Alexa 546 (red). Scale bars, 100 μm. **d** Cholesterol pull-down assay (*n* = 4). Cell lysates containing membrane fractions from stable Sf9 cells expressing WT-prestin-V5-GFP or 499-prestin-KI-V5-GFP were incubated with unconjugated control beads or cholesterol-conjugated beads, separated on SDS-PAGE, and probed with anti-V5. Both WT-prestin-V5-GFP and 499-prestin-KI-V5-GFP are pulled-down with cholesterol-conjugated beads but not with unconjugated beads

Although the concentration of HPβCD in various tissues (including the cochlear fluids) and in plasma is highest in the first two hours post-injection [14], the concentration of HPβCD and its elimination rate differ among different tissues. For example, HPβCD in plasma has a half-life (*t*_1/2_) of 1.0–1.6 h, while in brain *t*_1/2_ is 6.5 h [1, 48]. Since the half-life of HPβCD in the cochlea is not known, we directly addressed the contribution of prestin’s motile function by utilizing a prestin mutant, which carries the V499G/Y501H mutation located near the beginning of prestin’s C-terminal domain [58]. 499-prestin can be activated but only at highly depolarized potentials, making it dysfunctional at physiological membrane potentials [20]. Because mutant 499-prestin proteins localize to the lateral membrane [16, 58], the OHCs are normal in length and stiffness, which contrasts with the short, compliant OHCs in mice lacking prestin [29]. Using the 499-prestin KI mouse model, we asked whether non-motile prestin protects against HPβCD-induced OHC death. To this end, WT and 499 prestin-KI littermates received a one-time injection of either 4000 or 8000 mg/kg HPβCD when they were around three-weeks old. Cochlear samples were analyzed a week after injection as previously described [45]. As shown in Fig. 6a-c, 499-prestin-KI mice lose their OHCs just as WT, suggesting that prestin-based somatic electromotility does not contribute to the vulnerability of OHCs to HPβCD. Since WT-prestin directly interacts with cholesterol [45], we also examined whether mutant 499-prestin retains its ability to bind cholesterol. For these experiments, we established stable Sf9 cell lines that express WT or mutant 499-prestin to perform our in vitro cholesterol binding assay (*n* = 4). Equal amounts of cell lysates were incubated with cholesterol-beads and unconjugated beads. As shown in Fig. 6d, 499-prestin proteins expressed in Sf9 cells were pulled down by cholesterol-conjugated beads but not by unconjugated control beads, similar to the WT-prestin. Although prestin’s motile function does not appear to underlie the OHC’s susceptibility to HPβCD, the presence of prestin molecules that bind cholesterol may contribute to HPβCD-induced OHC death.

## Discussion

NPC1 disease affects the homeostasis of cellular cholesterol, which can have profound effects on cellular functions. Our evaluation of prestin protein expression and function in OHC of NPC1-KO mice was unaffected, except for the slight yet significant depolarization of the voltage operating point (V_pkcm_, Fig. 1g). This shift of V_pkcm_ in the depolarizing direction indicates a decrease in the amount of cholesterol in the OHC’s plasma membrane in NPC1-KO mice, which is plausible considering the altered cellular trafficking of cholesterol [49] and the high frequency threshold shifts in NPC1-KOs evident at weaning (Fig. 2). Although abnormal accumulation of cholesterol was observed in other cell types including spiral ganglion neurons and cells in the stria vascularis of NPC1-KO cochleae [23], OHC loss may be the predominant cause of threshold shifts in the basal high-frequency region in NPC1-KO mice (Fig. 3). High variability observed in this region of the cochlea in NPC1-KO mice may underlie individual variations in the progression of disease, as often noted in NPC1 patients [24].

It is generally understood that HPβCD helps reduce cholesterol accumulation in NPC1 disease by releasing lysosomal cholesterol into the cytoplasm [30, 39, 49]. However, in the cochlea, rapid OHC loss has been observed in animal models in a dose-dependent manner regardless of the mode of HPβCD administration [12, 13]. Since prestin is a lateral membrane protein HPβCD likely acts directly on plasma membrane cholesterol to confer its cytotoxic effect on OHCs. Curiously, some of the NPC1-KO mice receiving low-dose HPβCD (4000 mg/kg × 4) were resistant to the cytotoxic effect (Fig. 4a-b). This resilience may simply be a result of individual variations; however, it may also relate to the reduced plasma membrane cholesterol level in OHCs of NPC1-KO mice, revealed by NLC measurement (Fig. 1d, g). We do not fully understand what factors contribute to the variations observed in our data or the data in other publications. For example, multiple injections of 4000 mg/kg HPβCD did not cause permanent ototoxicity in FVB/NJ mice [13], while BALB/c mice lost a significant number of OHCs after 4 injections of 4000 mg/kg HPβCD. This information suggests that OHC sensitivity to HPβCD is influenced by strain background, which may also underlie the variations observed in human patients.

As one of the contributors to HPβCD-induced ototoxicity, prestin provides a potential molecular target for ameliorating unwanted side effect of HPβCD. Because salicylate is a small-molecule inhibitor of prestin’s motile function that was shown to have no adverse effect on NPC1-KO mice [43], we tested both oral and systemic administration protocols. Oral administration of salicylate failed to confer an inhibitory effect on prestin function (Fig. 5a). In contrast, direct systemic injection of salicylate increased ABR and DPOAE thresholds, indicative of prestin inhibition (Fig. 5b). However, it did not mitigate the ototoxic effects of HPβCD in either WT or in NPC1-KO mice. Thus, although potentially attractive, inhibition of prestin’s function by salicylate did not mitigate HPβCD-induced threshold shifts at the doses used in this report.

This conclusion was also corroborated by using 499-prestin-KI mice that express mutated prestin protein with virtually no motile function in vivo. Unlike prestin-KO mice, 499-prestin-KIs were as sensitive to HPβCD as WT (Fig. 6a-c), indicating that somatic electromotility per se is not directly linked to HPβCD susceptibility. The lateral wall of the OHC is highly specialized and consists of a trilaminate structure that includes an actin/spectrin-based cortical lattice and subsurface cisternae, linked to the prestin-embedded plasma membrane to form a distinct functional domain [26]. As an integral component of this trilaminate structure, prestin plays an important structural role [46]. Since prestin can directly interact with cholesterol [45], it is likely that the prestin-cholesterol interaction contributes to the stability of this specialized membrane domain. As 499-prestin retains its ability to bind cholesterol (Fig. 6d), it is similarly affected by HPβCD treatment as in WT.

Our study provides a detailed characterization of prestin expression and function in OHCs in the context of NPC1 disease. Although potentially promising, our study indicates that specifically targeting prestin did not provide protection of OHCs in response to HPβCD. Future efforts for the treatment of NPC1 disease should include effective drug delivery to avoid cochlear exposure, development of alternative small molecules that are more specific or non-toxic [4, 47], and gene therapy [7].

## Conclusions

- OHCs in NPC1-KO mice have normal prestin expression and motor function.
- HPβCD-induced ototoxicity is not dependent on prestin’s motile function.
- Salicylate, at the doses used in this report, does not mitigate HPβCD-induced ototoxicity in NPC1-KO mice.

## Additional file

Additional file 1: **Figure S1**. A. DP-grams for 2f1–f2 at high (L1 = L2 = 70 dB) stimulus levels for the same WT and NPC1-KO mice shown in Fig. 4A. Black lines show before and red lines after four weekly injections of 4000 mg/kg HPβCD treatments. Blue lines show responses of mice receiving a single administration of 8000 mg/kg HPβCD. B. DPOAE input-output functions of the same mice in A and B, showing responses for f2 = 12 kHz (f2/f1 = 1.2). **Figure S2**. Salicylate treatment of WT and NPC1-KO mice did not mitigate HPβCD-induced threshold shifts. A-B. DP-grams and input-output functions of Sal (O) and HP + Sal (O) groups from Fig. 5A, C-D are shown in A. WTs. B. NPC1-KOs. C-D. DP-grams of Sal (IP) and HP + Sal (IP) groups from Fig. 5B, C-D are shown. C. WTs. D. NPC1-KOs. (PDF 1136 kb)
