# Supplementary material for: The susceptibility of cochlear outer hair cells to cyclodextrin is not related to their electromotile activity
Source: Acta Neuropathol Commun. 2018 Sep 24;6:98. doi: 10.1186/s40478-018-0599-9 (PMC6151916; doi:10.1186/s40478-018-0599-9)
Supplement: Supplementary file 1 — Figure S1. A. DP-grams for 2f1–f2 at high (L1 = L2 = 70 dB) stimulus levels for the same WT and NPC1-KO mice shown in Fig. 4A. Black lines show before and red lines after four weekly injections of 4000 mg/kg HPβCD treatments. Blue lines show responses of mice receiving a single administration of 8000 mg/kg HPβCD. B. DPOAE input-output functions of the same mice in A and B, showing responses for f2 = 12 kHz (f2/f1 = 1.2). Figure S2. Salicylate treatment of WT and NPC1-KO mice did not mitigate HPβCD-induced threshold shifts. A-B. DP-grams and input-output functions of Sal (O) and HP + Sal (O) groups from Fig. 5A, C-D are shown in A. WTs. B. NPC1-KOs. C-D. DP-grams of Sal (IP) and HP + Sal (IP) groups from Fig. 5B, C-D are shown. C. WTs. D. NPC1-KOs. (PDF 1136 kb) [file 40478_2018_599_MOESM1_ESM.pdf]

Figure S1.

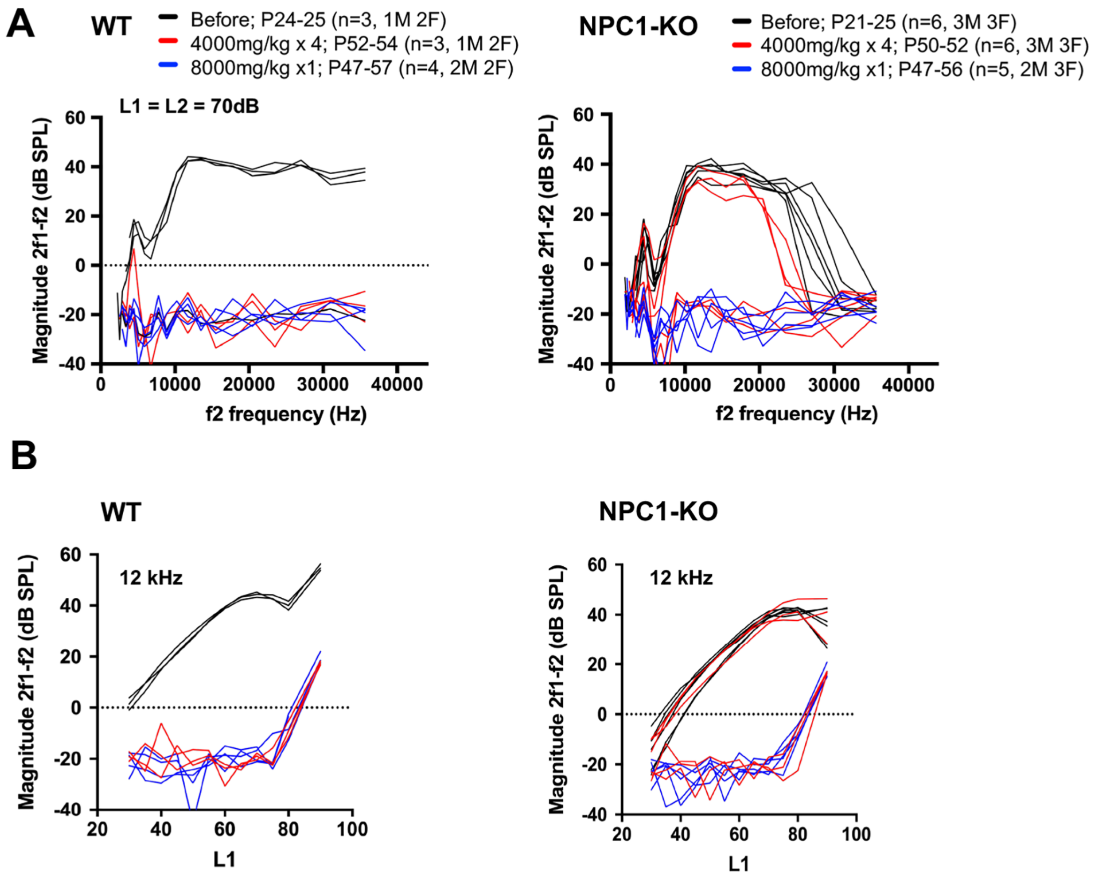

Figure S1. A. DP-grams for 2f1–f2 at high (L1=L2 =70 dB) stimulus levels for the same WT and NPC1-KO mice shown in Figure 4A. Black lines show before and red lines after four weekly injections of 4000 mg/kg HPβCD treatments. Blue lines show responses of mice receiving a single administration of 8000 mg/kg HPβCD. **B.** DPOAE input-output functions of the same mice in **A** and **B**, showing responses for f2=12 kHz (f2/f1=1.2).

Figure S2.

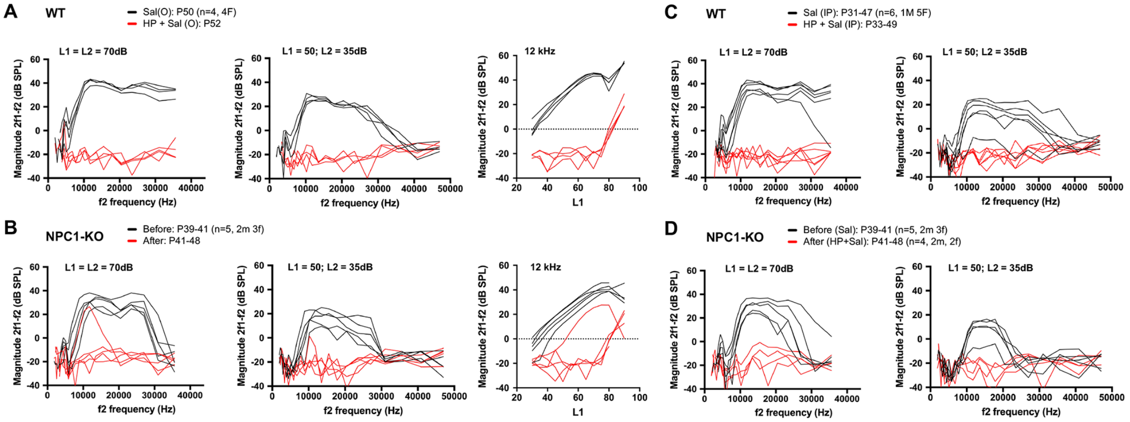

Figure S2. Salicylate treatment of WT and NPC1-KO mice did not mitigate HPβCD-induced hearing impairment. **A-B.** DP-grams and input-output functions of Sal (O) and HP + Sal (O) groups from Figure 5A, C-D are shown in **A.** WTs. **B.** NPC1-KOs. **C-D.** DP-grams of Sal (IP) and HP + Sal (IP) groups from Fig. 5B, C-D are shown. **C.** WTs. **D.** NPC1-KOs.
